# Abdominal shotgun trauma: A case report

**DOI:** 10.1186/1757-1626-1-34

**Published:** 2008-07-14

**Authors:** Konstantinos G Toutouzas, Andreas Larentzakis, Panagiotis Drimousis, Maria Riga, Dimitrios Theodorou, Stylianos Katsaragakis

**Affiliations:** 1 Surgical Intensive Care Unit, 1st Department of Propaedeutic Surgery, Hippokrateion General Hospital, Athens Medical School, University of Athens, Q. Sofias 114 av.,11527, Athens, Greece

## Abstract

**Introduction:**

One of the most lethal mechanisms of injury is shotgun wound and particularly the abdominal one.

**Case presentation:**

We report a case of a 45 years old male suffering abdominal shotgun trauma, who survived his injuries.

**Conclusion:**

The management of the abdominal shotgun wounds is mainly dependent on clinical examination and clinical judgment, while requires advanced surgical skills.

## Introduction

Firearm is the second leading mechanism of injury related death [1,2]. Additionally, abdominal shotgun wounds comprise a particularly lethal subset of shotgun injuries. Their mortality rate is three times greater than that of other shotgun wounds [3]. We present a case of shotgun trauma of the abdomen, with no lethal outcome.

## Case Presentation

An, otherwise, healthy 45 year-old male presented after sustaining an accidental shotgun trauma of the abdomen. It was a close-range injury. The entry wound was at the umbilical region. The patient was under hemodynamic instability. His heart rate was 122 beats per minute and his systolic blood pressure was 95 mmHg. He was tachypneic and had impaired mental status. During fluid resuscitation, the patient was immediately prepared for a diagnostic laparotomy.

**Figure 1:**
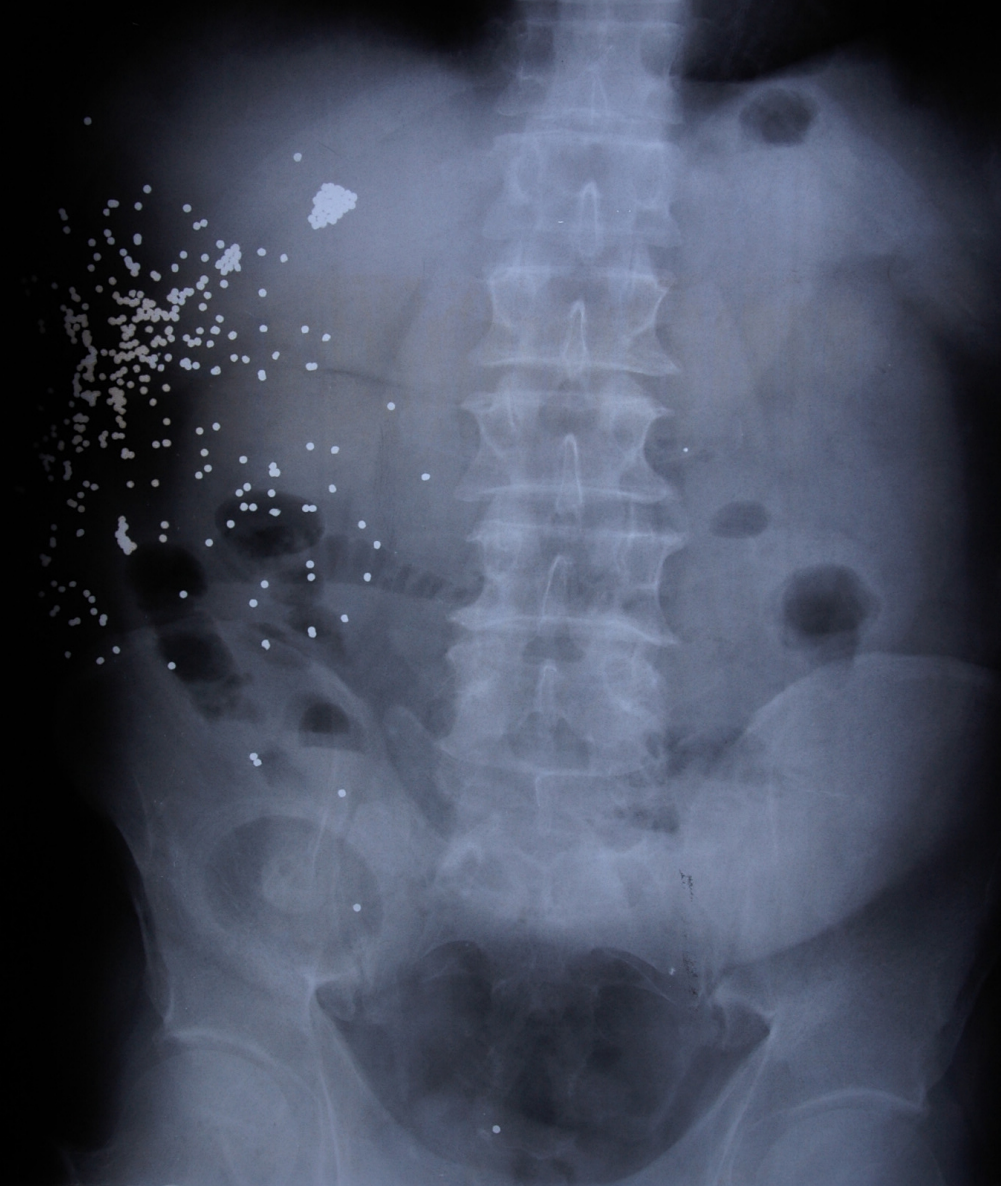
X-ray of the abdomen: This figure shows an X-ray of the abdomen, where the intra-abdominal pellets scatter is compatible with the intraoperative findings.

At the laparotomy, he was noted to have several injuries that included rapture of the rectus abdominis and deep fascia, a laceration of right hepatic lobe, a perforated gallbladder, a small non-expanding right-sided retroperitoneal haematoma, a total transection of the ascending colon near to hepatic flexure, several perforations of the ascending colon and of the proximal part of the transverse colon, a total transection of the most distal part of small intestine with large contamination and a bleeding laceration of the adjacent mesentery. Figure 1 shows an x-ray of the abdomen, where the intra-abdominal pellets scatter is compatible with the intraoperative findings.

The surgical repairs included hepatorrhaphy and use of topical haemostatic agent, right colectomy and anastomosis of the ileum with the transverse colon and open cholocystectomy. The peritoneal cavity was irrigated with warmed normal saline and drainaged, and the abdominal wall was completely closured. The intraoperative transfusion requirements were 6 units of packed red blood cells and 3 units of fresh frozen plasma.

After the operation, the patient was admitted in the intensive care unit (ICU). The 4^th ^postoperative day he was transferred out of the ICU. His total in-hospital stay was 18 days.

One year later, the patient was admitted to hospital in order to undergo an elective operation for an abdominal wall hernia repair. He was discharged the 6^th ^postoperative day.

## Discussion

Shotguns can cause a wide variety of trauma regarding the type and severity of the injury. The main factors affecting the severity of these injuries are the type of the gun and pellets, and the weapon-victim distance. Several wound classification systems have been proposed in order to predict the significance of internal injuries and to facilitate the clinical decision-making on selecting patients for emergency operation or non-operative management. Sherman et al. [4] classified shotgun wounds into three types based upon distance and penetration. Glezer et al. [5] redefined Sherman's groups by pellets spread, into three types of trauma patients, as a more useful classification system in determining the management of patients. Nevertheless, the comparison of the clinical examination with a new classification system based on the number of body areas involved, by Velmahos et al [6], concluded that clinical examination is the most reliable tool to guide the management of trauma patients suffering shotgun wounds, and that classification systems do not reliably predict the presence of significant internal injuries. Also, the clinical judgement remains the best available predictor of the need for laparotomy, even when being compared with the initial clinical status using the Emergency Room Trauma Score (ER TS), which is calculated from Glasgow Coma Scale, systolic blood pressure, respiratory rate and expansion, and capillary refill. [3]. An interesting subset of patients is that with penetrating abdominal shotgun wounds and stable vital signs, as these patients have a more difficult decision making. In these cases, laparoscopy, when not contraindicated, seems to be a safe and accurate approach, in order to effectively eliminate non therapeutics laparotomies [7].

In the case presented, the need for laparotomy was indicated mainly because of the hemodynamic instability of the patient, as it was evaluated by patient's vital signs, while parameters as the area involved and the pellets scatter were used just to guide the clinical estimation about the intra-abdominal structures involved.

## Conclusion

The spectrum of severity of the abdominal shotgun injuries is vast. This subset of trauma remains a particularly difficult challenge for the trauma surgeon, not only because of the advanced surgical skills needed, but also because the clinical judgement is the main tool of the decision making regarding the operative or non-operative management of these patients.

## Consent

Written informed consent was obtained from the patient for publication of this case report and accompanying images. A copy of the written consent is available for review by the Editor-in-Chief of this journal.

## Competing interests

The authors declare that they have no competing interests.

## Authors' contributions

KGT contributed to the management of the case, conception and design of the manuscript and revised it critically. AL contributed to the conception, design and drafting of the manuscript. PD contributed to the acquisition of data and to the drafting of the manuscript. MR contributed to the analysis and interpretation of data and to the drafting of the manuscript. DT contributed to the conception and design of the manuscript and revised it critically. SK supervised the management of the case, obtained written consent, contributed to the conception and design of the manuscript and revised it critically. All authors read and approved the final manuscript.
